# Senior Centers and LGBT Participants: Engaging Older Adults Virtually in a Pandemic

**DOI:** 10.1093/geroni/igab046.1139

**Published:** 2021-12-17

**Authors:** Suzanne Marmo, Manoj Pardasani, David Vincent

**Affiliations:** 1 Sacred Heart University, Fairfield, Connecticut, United States; 2 Adelphi University, Garden City, New York, United States; 3 SAGE, NEW YORK, New York, United States

## Abstract

Upon the outbreak of COVID-19, recommendations to cease all non-essential in-person social services were mandated across the United States to prevent transmission to non-infected individuals. As a result, approximately 96% of all senior centers in the United States were closed to in-person programming (National Council on Aging, 2020). LGBT older adults in particular were at higher risk of isolation and declines in overall health as they were more likely to live alone, experience loneliness or have less immediate family support systems when compared to non-LGBT older adults (Yang, Chu & Salmon, 2017). The purpose of this presentation is to explore how LGBT older adult participants in senior centers transitioned to virtual programming during the pandemic. Using a risk-resiliency theory framework, the purpose of this presentation is to share the impact of virtual programming on the health and well-being of LGBT community-dwelling older adults. An exploratory, cross-sectional study was conducted utilizing an online survey to understand their needs, concerns and experiences. Participants reported a relatively easy adaptation to technology, steady participation in programs and services, satisfaction with virtual senior center programming and a consistent sense of engagement with their peers. Higher levels of engagement with senior center programs were associated with greater perceptions of social support. Additionally, stronger perceptions of social support and participation in exercise and fitness programming were associated with higher life satisfaction and lesser symptoms of depression and anxiety. Strategies for outreach, engagement and service provision will be presented.

